# “When you live in a colony… every act counts”: Exploring engagement in and perceptions of diverse anti‐colonial resistance strategies in Puerto Rico

**DOI:** 10.1111/bjso.12808

**Published:** 2024-10-09

**Authors:** Carmen Marazzi, Johanna Ray Vollhardt

**Affiliations:** ^1^ Clark University Worcester Massachussetts USA

**Keywords:** collective action, colonialism, coloniality, cultural resistance, everyday resistance, oppression, prefigurative politics, Puerto Rico, resistance

## Abstract

While social psychology has contributed much to our understanding of collective action, other forms of resistance are understudied. However, in contexts of long‐standing oppression—such as ongoing colonialism—and past repression of liberation struggles, other resistance strategies are important considering the constraints on overt, collective action in such contexts. This paper reports findings from an interview study in Puerto Rico (*N* = 22) exploring anti‐colonial resistance. We analysed participants' own resistance, future preferred strategies, and descriptive norms of other ingroup members' resistance. Through thematic analysis, we identified six distinct forms of anti‐colonial resistance. Notably, none of the participants reported participating in collective action. Instead, participants engaged in different forms of symbolic everyday resistance to preserve a positive, distinct cultural identity, and raise critical consciousness of the group's oppression. Additionally, more tangible resistance strategies included staying on the land and building independent economies. Overall, this study illustrates the importance of considering a more comprehensive set of resistance strategies in contexts of long‐standing colonial oppression to recognize oppressed groups' agency and provide a better understanding of how people undermine destructive power.

## INTRODUCTION

In the summer of 2019, Puerto Ricans came together to demand the Governor's resignation through massive protests, highway shutdowns, and creative public demonstrations (Henríquez, 2019). These efforts were successful, demonstrating the power of collective action when calling for a demand like the resignation of then‐Governor Rosselló. Puerto Ricans have also used demonstrations, disruptive measures, and encampments to demand the end of gender‐based violence (Pérez Pintado, 2019), the shutdown of the US military bases in Vieques and Culebra (Schils, 2011; Walsh, 2001), to end budget cuts to public universities (Robles, 2017; Rosa, 2016), and in solidarity with Palestine (Awartani, 2017; Rios Mace, 2024). Yet, when it comes to demanding liberation or independence from US colonial rule over Puerto Rico, these tactics are not taken up at‐large in the present. This may be, in part, because past independence movements were severely repressed and unsuccessful (Atiles‐Osoria, 2016; Silén, 1971).

Despite this, anti‐colonial resistance is sometimes mentioned in protests about other causes. For example, some signs at the 2019 protest demanding the governor's resignation also articulated an anti‐colonial demand: “¡Ricky renuncia, y llévate a la Junta!” (Ricky, resign, and take the [Fiscal Control Board] with you).^1^ This suggests that anti‐colonial sentiment may be more widespread than the lack of overt protests around this issue makes it seem, and that it is necessary to examine other forms of anti‐colonial resistance beyond prototypical collective action (i.e., protest; Rosales & Langhout, 2020; Vollhardt et al., 2020) to avoid portraying oppressed groups' as passive and to better understand the psychology of resistance. Therefore, the present paper explores how Puerto Ricans engage in anti‐colonial resistance: which resistance strategies people choose, perceive other group members to engage in, and which resistance strategies they believe should be enacted for future steps towards liberation.

### The colonial history and present of Puerto Rico

Puerto Rico today is a colonial possession of the United States, and its society is the product of over 500 years of colonialism (Meléndez‐Badillo, 2024). It was one of the first sites of colonial oppression in the Americas in 1493, under Spanish rule. After the United States invaded Puerto Rico in 1898 during the Spanish‐American War, Puerto Rico became an “unincorporated territory” of the United States. With the creation of the *Estado Libre Asociado* (ELA; Commonwealth of Puerto Rico), Puerto Ricans were permitted to write their own constitution and elect their own leaders. However, laws like PROMESA and the Jones‐Shafroth Act give the US government control over Puerto Rico in matters of trade, defence, governance, and citizenship. Additionally, assimilation has been encouraged by repressing Puerto Rican identity (Alejandrez & Liberato, 2022). For example, English was imposed as the primary language in the early stages of US colonial rule (Pausada, 1999), and the 1948 Gag Law criminalized owning or displaying a Puerto Rican flag, singing the Puerto Rican anthem, or expressing support for independence. Furthermore, independence movements were violently repressed by the United States through the political surveillance and criminalization of its leaders and any potential supporters (Atiles‐Osoria, 2016; Poitevin, 2000). In the light of this repression, it is necessary to examine what other strategies of resistance may be used in this context.

### Conceptualizing resistance

Resistance involves action and opposition (Hollander & Einwohner, 2004). In contexts of oppression, this entails challenging the group's subordination and undermining the oppressor's goals and power (Haslam & Reicher, 2012; Vollhardt et al., 2020). However, “action” is broadly defined and does not need to be overt. It can be subtle and symbolic (Rosales & Langhout, 2020; Scott, 1985), and involve critical consciousness (Freire, 1970) where people reflect on the nature of oppression and act on this awareness to counter it.

#### Collective action – and the need to expand the scope of social psychological work on resistance

The prototype of resistance that is most studied in social psychological research is collective action through protests (Thomas et al., 2022; van Zomeren et al., 2012; Vollhardt et al., 2020). Collective action is commonly defined as actions that “individuals undertake as group members to pursue group goals such as social change” (van Zomeren et al., 2018, p. 122). More recently, social psychologists have begun to consider the influence of repression and risks of organized, overt collective action that may change the psychological processes of collective action (e.g., Ayanian et al., 2021) and which resistance strategies people choose (Rosales & Langhout, 2020).

Beyond social movements and protests, there are other forms of collective action that are understudied in social psychology, such as prefigurative politics (Cornish et al., 2016). This involves social change “here and now” by creating “local and collective structures that anticipate the future liberated society” (Boggs, 1977, p. 103). For example, community‐based groups can come together to address community members' needs that are not met by official institutions, and empower people (e.g., Dutt, 2018). These strategies of autonomous organizing have been crucial for community survival in Puerto Rico in the aftermath of Hurricane María (Ortiz Torres, 2020; Serrano‐García, 2020), for example by organizing mutual aid such as community kitchens, distribution centres, health clinics, shelters, and solar generation networks. These grassroots strategies challenge systemic violence and resist against the suffering that the official systems create.

Both the growing social psychological work on collective action in contexts of repression (Ayanian et al., 2021) and work examining resistance more broadly (Rosales & Langhout, 2020; Vollhardt et al., 2020) suggest the importance of expanding how we understand and study resistance: for example, by taking the context of collective action into account more (Saavedra & Drury, 2019); by diversifying the scope of collective action strategies that are considered (e.g., prefigurative politics), and by examining different forms of resistance as well as how these different tactics relate to overt collective action (Orazani & Teymoori, 2024). This includes resistance strategies that need not be overt or collective but allow individuals to resist in “tight spaces of oppression” (Rosales & Langhout, 2020)—yet are rarely considered given the perceived importance of collective action to enact social change. Expanding the scope of resistance strategies that are studied in social psychology beyond overt collective action through protests and petitions is important for theoretical, ethical, and practical reasons: First, it recognizes oppressed groups' agency and range of resistance actions and helps avoid interpreting a lack of overt, anti‐colonial collective resistance as passivity or an indicator of internalized inferiority and coloniality (Bulhan, 2015; David & Okazaki, 2006) linked to colonial system justification (Rivera Pichardo et al., 2022). Second, it allows us to work towards a broader theoretical conceptualization of the social psychology of resistance and social change (Sweetman et al., 2013). Some of this broader theorizing about the psychology of resistance has been proposed in work on everyday and psychological resistance, to which we turn next.

#### Everyday resistance

People also resist oppression and undermine destructive power in their daily lives through everyday resistance (Johansson & Vinthagen, 2019; Scott, 1985). These strategies include mundane, often subtle actions that individual members of the group can take up without formal organization (Rosales & Langhout, 2020). These strategies are diverse and understudied in social psychology.

One form of everyday resistance is psychological resistance, where people oppose dominance by determining the psychological meaning of their group's disadvantage for themselves (Leach & Livingstone, 2015). In contexts of colonialism and other asymmetric power relations, this could involve rejecting colonial mentality or internalized inferiority (Bulhan, 2015; Capielo Rosario et al., 2019), combating negative stereotypes of the group (Suyemoto et al., 2022), or using humour to subvert and exert some control over the relations with the dominant group (Dobai & Hopkins, 2020).

Where the group's cultural identity is also threatened, ingroup culture is crucial for (everyday) resistance (Livingstone et al., 2023). This is particularly relevant for colonialism and coloniality, which include domination through culture and identity (Bulhan, 2015). Accordingly, maintaining culture is a resistance strategy against colonial oppression. Cultural resistance involves using the group's shared meanings and symbols to contest dominant discourses and power structures (Duncombe, 2007). While individuals practice cultural resistance through language (Livingstone et al., 2023), art, and music in their daily lives, it is also often part of collective resistance, and protest symbols can mobilize social change (Awad & Wagoner, 2020).

#### Group resistance norms and desired future resistance strategies

To understand which of these many different resistance strategies people consider to be relevant in a given context, it is important to examine not just what people themselves do but also which resistance strategies they see other group members using. Generally, people consider their group's social norms to understand a situation and respond to it appropriately (Cialdini & Goldstein, 2004). The social identity model of collective action posits that norms of participation within the ingroup, referred to as “instrumental support”, influence willingness to engage in collective action (van Zomeren et al., 2004). Accordingly, a survey among a representative sample of Palestinians in the Occupied Palestinian Territories found that perceived community norms of resistance predicted motivation for nonviolent resistance (Penić et al., 2024). Presumably, norms are also relevant for other forms of resistance. Seeing which resistance strategies are normative in their communities may shape which specific resistance strategies people prefer or see as feasible.

Which resistance strategies people think the ingroup should pursue in the future is also important to consider—in addition to perceived descriptive norms in the present—to gain a more complete understanding of the psychology of resistance in a given context. Imagining a liberated future society and dreaming of different possibilities for one's group outside of oppression is central for resistance in the present (Badaan et al., 2022; Mosley et al., 2020; Yosso, 2005). Such freedom dreams have informed oppressed groups' political movements throughout history (Kelley, 1993). This imagining may entail not just the endpoint of liberation but also the steps needed to get there. It may therefore inform which forms of resistance are currently practised to work towards the next desired resistance strategy.

### Overview of research questions

Overall, people may resist colonial oppression in subtle, informal, and less organized ways than the collective action strategies commonly studied in social psychology (Cornish et al., 2016; Rosales & Langhout, 2020; Vollhardt et al., 2020). These diverse resistance strategies are important to examine to recognize oppressed groups' agency and avoid mischaracterizing seeming passivity to oppression, as well as to inform theorizing and a more comprehensive conceptualization of the psychology of resistance that goes beyond prototypical forms of protest.

The Puerto Rican context is ideal for examining the confluence and diversity of resistance strategies, given the history of violent repression against independence movements (Atiles‐Osoria, 2016) and the pervasive nature of colonization and coloniality in all spheres of life—“from occupation of land to occupation of being” (Bulhan, 2015, p. 239). Therefore, the present paper examines the following research questions: (1) Which theoretically distinct, anti‐colonial resistance strategies do Puerto Ricans take up against US colonialism? (2) Which resistance strategies are seen as normative within the ingroup? (3) Moreover, which desired, future resistance strategies are expressed as part of the psychology of anti‐colonial resistance?

## METHOD

### Sample

The sample included 22 Puerto Ricans (*M =* 40.5 years old, *SD* = 20.10), living in different parts of Puerto Rico. Most self‐identified as women (*n* = 13), others as men (*n* = 9). Ideologically, most participants supported independence (*n* = 10), others supported Puerto Rico becoming an official state of the United States (i.e., “statehood”, *n* = 6), or maintaining the status quo (*n* = 3). Three participants supported an anti‐colonial option beyond independence (e.g., free association). Participants were highly educated, with all having at least some university education. Considering the complexity of racial identification in Puerto Rico (Godreau & Bonilla, 2021), we asked participants to indicate their race/ethnicity with an open‐ended question. Most (*n* = 12) identified as White, others as Trigueño/a (*n* = 3), Black/Afro‐descendent (*n* = 2), Latine or Hispanic (*n* = 7), or they used a non‐racial description (see Table 1 for more detailed sample characteristics).

**TABLE 1:** Participant demographics.

ID	Age	Gender	Race/ethnicity	SES/class	Education	Occupation	Municipality	Status preference
1	55	Woman	White, Puerto Rican	Upper middle	Bachelor's degree	Publicist	San Juan	Status quo
2	62	Woman	White, Latina	Upper middle	Doctorate degree	Artist, educator	San Juan	Independence
3	27	Woman	Puerto Rican	Upper middle	Bachelor's degree	Student, Journalist	San Juan	Independence
4	73	Woman	Trigueña	Middle	Doctorate degree	Psychologist	San Juan	Independence
5	66	Man	Human	Upper middle	Doctorate degree	Retired, Activist	Cabo Rojo	Independence
6	27	Woman	Hispanic	Middle	Master's degree	Non‐profit	San Juan	Anti‐colonial
7	63	Woman	White, Hispanic	Middle	Bachelor's degree	Administrator	San Juan	Statehood
8	51	Man	White, Caribbean	Middle	Bachelor's degree	Manual labor	Ponce	Statehood
9	79	Woman	White, Puerto Rican	Upper middle	Master's degree	Retired, teacher	San Juan	Statehood
10	56	Woman	White	Middle	Some college	Retail	Vieques	Statehood
11	21	Woman	Trigueña	Lower middle	Some college	Medical student	Caguas	Independence
12	24	Man	Black, Latino	Middle	Some college	Student/non‐profit	Toa Baja	Independence
13	22	Man	White	Low/in poverty	Some college	Student	Aguas Buenas	Status quo
14	23	Woman	White	Lower middle	Some college	Student	Morovis	Independence
15	19	Woman	White, Latina	Middle	Some college	Student	Isabela	Independence
16	22	Woman	Puerto Rican, Latina	Lower middle	Some college	Student	Las Piedras	Independence
17	29	Man	Afro‐descendant	Lower middle	Master's degree	Teacher	Barceloneta	Independence
18	25	Woman	Hispanic	Middle	Bachelor's degree	Student	Guayanilla	Status quo
19	25	Man	White, Caribbean	Lower middle	Bachelor's degree	Writer/Editor	Isabela	Anti‐colonial
20	22	Man	White, Caribbean, Puerto Rican	Middle	Some college	Student	Barceloneta	Anti‐colonial
21	59	Man	Trigueño	Low/in poverty	Associate's degree	Retired accountant	Trujillo Alto	Statehood
22	41	Man	White	Middle	Bachelor's degree	Teacher	San Juan	Statehood

### Procedure

The study was conducted in 2022 and early 2023. Participants were recruited through convenience sampling, via social media and flyers in public places across the islands. Potential participants were informed that the study was about their perception of the relationship between Puerto Rico and the United States. The interviews lasted 30 min to 2 h and took place in Spanish over the phone or Zoom, by participants' choice and due to the COVID‐19 pandemic. After obtaining informed consent, interviews were conducted by the first author and audio recorded. While the interview was a broader study about perceptions of the US‐Puerto Rican relationship (see online Appendix S1 for full interview schedule), the present analysis focuses on the questions about resistance, specifically: “What are the ways in which Puerto Ricans show opposition to [participant's term for *PR‐US relationship]*?”, “Do you think these strategies have been effective?”, “What should ideally be done to address the issue of [*PR‐US relationship]*?”, “Do you consider yourself an activist?” Notably, this wording avoided prompting participants to use the terms “colonialism” or “resistance”, instead using their preferred terms. Additionally, our analysis included any answers that mentioned resistance without it being directly prompted (e.g., we found this in response to the question “Can you describe what it means to be Puerto Rican?”). Following the interview, participants were debriefed and offered compensation for their time. The recordings were transcribed by the first author and five Puerto Rican undergraduate research assistants.

### Analytic procedure

We used reflexive thematic analysis to identify patterns of meaning in the data that address our research questions (Braun & Clarke, 2022). We used an inductive approach to identify a broad range of different resistance strategies without imposing expectations on what we would find. Then, we refined, organized, and named most of our themes deductively, drawing on the literature to name different resistance strategies (e.g., psychological resistance; Leach & Livingstone, 2015) and subthemes (e.g., future desired strategies; Kelley, 1993) whenever possible and distinguish other relevant theoretical dimensions (e.g., individual vs. collective resistance; Tajfel & Turner, 1986).

The first author first read and familiarized herself with the data, segmenting quotes from the interviews that were relevant to our research questions. Resistance strategies that were brought up but not meant to resist US colonialism were omitted from this analysis. Initial codes summarized the essence of each segment. Subsequently, these segments were grouped to create themes capturing the different resistance strategies expressed. We also noticed that different themes were often represented on different levels: as resistance strategies participants personally engaged in, described other group members were doing, or what they thought the ingroup should engage in in the future. We decided this was a theoretically and analytically interesting distinction, and accordingly developed subthemes to capture these different levels of the resistance strategies.

The first author then translated the quotes from each initial theme and subtheme into English so that the second author could review them and serve as an analytic auditor (de Kleijn & van Leeuwen, 2018). Both authors refined the themes together (for example, moving quotes that fit a different theme better, or collapsing themes that had few quotes and were conceptually related). The analysis was also informed by discussions with the undergraduate research team.

### Reflexivity

To engage in a process of reflexivity (Braun & Clarke, 2022), the first author documented how the research process was shaped by her perspective and social position. The first author is a white, bicultural Puerto Rican born and raised in San Juan. While her analysis is informed by her insider perspective as a Puerto Rican, her bicultural background and current residence in the US mainland also brings an outsider perspective to this analysis (see Ademolu, 2023). Her desire for the liberation of Puerto Rico and of all peoples under colonial oppression shaped the current research questions. The second author is white, has US and EU citizenship, and has a multi‐cultural background. Her analysis is informed by her research on collective victimization and resistance across the globe, and her political solidarity with anti‐colonial liberation struggles that shape an interest in understanding the diversity of resistance tactics in such contexts.

Among the Puerto Rican undergraduate research assistants at the authors' university who transcribed and provided insights into the analysis, two grew up in Puerto Rico and the other three in the US mainland. The group was multiracial and held differing political perspectives on Puerto Rico's status.

## FINDINGS

Based on what participants described as opposition to the US‐Puerto Rican relationship, we identified six themes capturing theoretically distinct anti‐colonial resistance strategies: (1) psychological resistance, (2) critical consciousness‐raising, (3) symbolic and cultural resistance, (4) staying on the land despite struggle and hardship, (5) prefigurative politics, and (6) protests. The first four themes capture different types of everyday resistance (Vinthagen & Johansson, 2013), while prefigurative politics and protests are forms of collective action (Cornish et al., 2016; Thomas et al., 2022). These strategies included both individual and collective levels, which are not mutually exclusive. For example, countering negative identity portrayals at the individual, identity‐focused level can also be understood as targeting the collective problem of coloniality (Bulhan, 2015).

**FIGURE 1:**
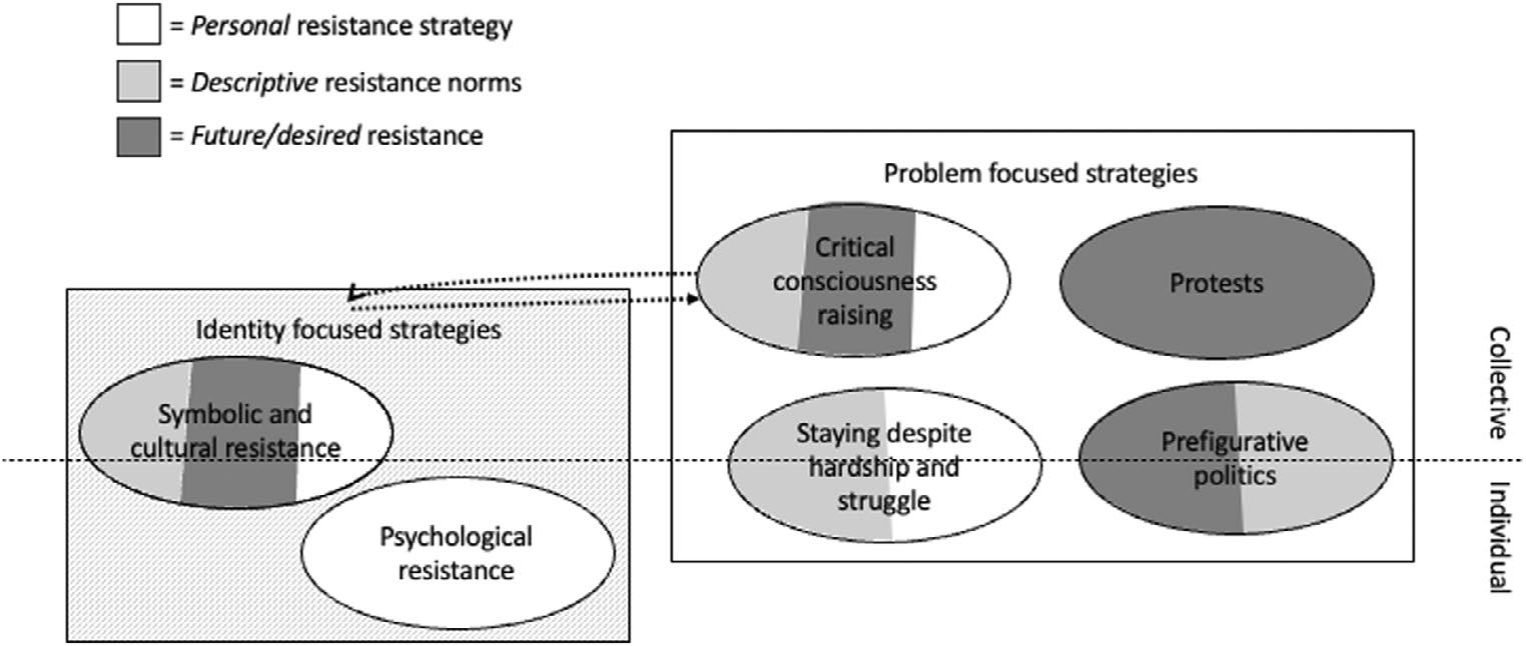
Thematic map of resistance strategies and their theoretical dimensions.

Additionally, we examine how participants discussed these strategies across three dimensions: (a) *personal* resistance strategies, or strategies that participants themselves engage in; (b) *descriptive norms*, or anti‐colonial resistance strategies that participants observed among the ingroup; and (c) *desired, future resistance strategies*, or resistance strategies that participants believed the group could take up in the future. Because these dimensions are not consistently represented, we specify which dimensions were found within each theme (see Figure 1).

### Psychological resistance

Psychological resistance is when individuals resist oppression by determining the meaning of their group's disadvantage (Leach & Livingstone, 2015). Presumably because of this strategy's individual nature, participants only described how they themselves express critical awareness of colonial oppression (Freire, 1970). Specifically, participants described resistance through questioning and countering colonial narratives. For example, Puerto Rican history and literature courses include texts that essentialize Puerto Ricans' psychology and personality to negative stereotypes (Marqués, 1977; Pedreira, 1979; see Montijo, 1988 for a critique). A 22‐year‐old female independence supporter reported refuting this master narrative by avoiding these texts:

I know that there are essays about the fragile Puerto Rican, lazy. I didn't want to read them. They seem very self‐deprecating. The Puerto Rican can think and say that “Oh, we are very fragile” (…) and I'm like “oh, ok”… you know that in Puerto Rico there's been years of colonization and all that. How do you expect to grow when they put such a heavy hand over you? It feels very unjust and self‐deprecating how one can think of oneself. (*Participant 16*)

This quote illustrates two distinct processes. First, this participant recognizes the psychological, symbolic threat these texts could pose to her collective self‐esteem, and counters it through avoidance and by reframing the meaning of the identity to protect herself from harm. Thereby, she opposes what the psychological literature describes as “internalized oppression” and often assumes to be pervasive (David, 2014). Second, she recognizes how these stereotypes reinforce colonial ways of being (Bulhan, 2015), which can stunt potential progress as a nation. Support for US colonial rule is often sustained by self‐blame for Puerto Ricans' problems (Varas‐Díaz & Serrano‐García, 2003) rather than focusing on the structural colonial policies that hinder growth. Therefore, rejecting the normalization and internalization of negative stereotypes taught through the official curriculum, and shifting to external attributions for the group's problems as this participant does (“*they* put such a heavy hand”), is a powerful form of psychological resistance.

Beyond rejecting negative stereotypes, people can also shape the meaning of their identity by highlighting the positive, symbolic significance of events pertaining to their ingroup. For example, a 22‐year‐old male status quo supporter said about Puerto Rican identity:

…what's important is when something happens with someone who is famous in the sense that you're with your community waiting to see if they get a prize, like with tennis with Monica Puig, the one that ran, or even Miss Universe, that you feel part, right, that you are a country, and you are not subordinated even with the situation that exists. (*Participant 13*)

This participant mentions several events where Puerto Rican athletes or citizens were internationally recognized with competitive awards. This fulfils a symbolic need for national, independent representation despite the colonial relations and can give Puerto Ricans a sense of pride, as the participant suggests. Although it does not change the ingroup's material conditions, the participant emphasizes that this achievement refutes their subordination in other ways, through a positive identity—in line with Haslam and Reicher's (2012) definition of resistance as “the process and act of challenging one's subordinated position in a given social system” (p. 155). Social identity theory suggests that social creativity, or modifying perceptions of the ingroup's standing and changing the social comparison to be more favourable, is distinct from collective action and thereby from resistance (Tajfel & Turner, 1986). However, because colonial oppression dominates “the total being of the colonized–economically, culturally, socially, and psychologically” (Bulhan, 2015, p. 253), reclaiming the psychological meaning of the oppressed identity is a crucial way of resisting such total control over one's being (Gramsci, 1971; Memmi, 1965). It is an important future research question whether and how everyday resistance, including psychological resistance and “oppositional identities” (Orazani & Teymoori, 2024), could mobilize collective resistance in the longer term (see Rosales & Langhout, 2020).

### Critical consciousness‐raising

Related to psychological resistance, several participants discussed critical consciousness‐raising—that is, encouraging other ingroup members to develop critical awareness of colonial oppression, which is a basis for other forms of anti‐colonial resistance. The salience of this theme is unsurprising, as the notion of critical consciousness‐raising originates from Latin American scholars (Freire, 1970; Martín‐Baró, 1994). Most participants who expressed this theme were educators or students, whose daily lives involve sharing knowledge. While this resistance strategy was represented across all three dimensions we considered, due to space constraints we only elaborate on two of them (see Appendix S2 for sample quotes for all dimensions).

#### Critical consciousness‐raising as a personal resistance strategy

Education has been a tool of colonization to promote colonialities of being (Bulhan, 2015), which participants mentioned a lot. Therefore, education needs to be purposeful to counter colonial mentalities. For example, a 66‐year‐old male independence supporter described the need for consciousness‐raising efforts through his work as a popular educator for an anti‐colonial group:

I feel inclined to work on the education process but through the perspective of popular education in that we consider that our people know. Our people aren't dumb, they aren't ignorant, (…) but not necessarily all of our people have the tools to interpret, really, the oppression that we are being submitted to. (*Participant 5*)

This quote illustrates the relation between the current and the previous theme, suggesting that psychological resistance (countering negative stereotypes of Puerto Ricans) may be the basis for engaging in critical consciousness‐raising as well as one of its goals. In addition to popular education, consciousness‐raising efforts participants reported occurred within their interpersonal relations (e.g., questioning their loved ones' beliefs) and respective occupations (e.g., journalism). Some examples of this theme further illustrate that in this context everyday resistance can entail more organized, collective resistance (e.g., popular education organized by a group). Thus, this finding suggests that everyday resistance and collective resistance are not mutually exclusive.

#### Critical consciousness‐raising as a desired, future resistance strategy

Participants also discussed critical consciousness‐raising as a desired, future resistance strategy. For several participants, this involved continued consciousness‐raising efforts in their professions (e.g., journalism, activism) and interpersonal relations. Others believed these efforts should also involve addressing shortcomings of the current practices. For example, a 22‐year‐old male anti‐colonial supporter highlighted the importance of using venues and cultural tools accessible to all Puerto Ricans to bring people together and raise critical consciousness:

I had a project back then but those who are taking it up now are making it cooler (…). It would summon people to educate themselves in *chinchorros*. This is because you need to use culture. That is what I see because if you want a change you need to appeal to culture and educate through culture. (*Participant 20*)

This participant illustrates a space (*chinchorros* are small local establishments serving food and drinks) that could be used for dialogue outside of academic institutions. This is important given the critique of past independence movements belonging to an intellectual working‐class elite who often paternalized and erased workers who did not fit the white and male working‐class identity of *puertorriqueñidad* (Meléndez‐Badillo, 2019). The maintenance of racialized, gendered hierarchies is linked to coloniality and perpetuates oppression (Quijano, 2007). Therefore, to further cultivate critical consciousness as a future grassroots resistance strategy, these power dynamics need to be avoided by being in spaces where Puerto Ricans will not be marginalized, as this participant quote implies.

### Symbolic and cultural resistance

In addition to using cultural tools to engage in critical consciousness‐raising, symbolic and cultural resistance was also a theme in its own right. Cultural resistance is defined as the use of culture to resist and/or change the dominant political, economic, and social structures (Duncombe, 2002). While the previous themes discuss countering negative stereotypes and deficit narratives of Puerto Ricans within oneself and encouraging this critical consciousness in others, the present theme more broadly centres preserving a distinctive Puerto Rican cultural identity that counters US (colonial) ways of being, and thereby challenges US cultural dominance. Notably, this intention (opposing US colonialism through symbolic and cultural means) is decisive, and not all cultural activity constitutes resistance (Duncombe, 2002). While this theme was represented across all three dimensions, given the overlap in symbolic tools (e.g., language, identity) discussed we only elaborate on examples from two of the dimensions.

#### Symbolic and cultural resistance as a personal resistance strategy

A primary source of resistance for Puerto Ricans has been through strong ingroup identification. For example, a 29‐year‐old male independence supporter stated that “being Puerto Rican is what I understand to be more of an act of resistance” (*Participant 17*). While group identity predicts collective action (e.g., van Zomeren et al., 2012), this quote suggests that resistance itself can be a form of identity content (Reicher et al., 2006)—where the distinctions between cultural (and other forms) of resistance and ingroup identity are fluid (see also Livingstone et al., 2023). Notably, this resistance strategy overlaps to some extent with, but is also distinct from, psychological resistance centred on the meaning of the ingroup's disadvantage. Instead, merely asserting the identity is an act of anti‐colonial resistance in this context of past cultural and national repression.

Language is also an important tool of symbolic, cultural resistance in Puerto Rico. Despite the imposition of English, a 27‐year‐old woman independence supporter states that the creolization of Spanish and English enrichens the language spoken by Puerto Ricans while also distancing oneself from former and current colonizers and challenging their dominance. Moreover, in this multilingual context with clear power differences attached to language, how languages are spoken can be a symbolic, everyday act of resistance. For example, a 73‐year‐old woman (independence supporter) who frequently moved between Puerto Rico and the US mainland stated:

I have studied English all my life, and you hear a very strong accent. For me, it has always been a way (…) to defend my identity, of the little that's left. And I do not want to leave my accent. I know people who [say] “wow, they have no accent and they put effort in”. For me, all the contrary. I put effort into making it feel like I don't belong there, and that I'm from here. (*Participant 4*)

Considering this participant's age and lived experiences of more overt acts of repression of Puerto Rican identity and culture, the choice to maintain an accent when speaking English is both an act of defiance against forced assimilation, and an act of establishing distinctiveness in the US mainland. Overall, this theme captures the use of cultural tools to maintain a distinctive group identity to counter colonial domination of culture and identity (Bulhan, 2015), while the previous theme of psychological resistance is about challenging the negative meaning of the disadvantage attached to the ingroup's identity. While related, these two themes are therefore distinct.

#### Symbolic and cultural resistance as a descriptive resistance norm

In describing norms of symbolic and cultural resistance, participants included preserving Puerto Rican culture more generally in addition to specific identity‐ and language‐based resistance strategies. For example, a 23‐year‐old woman independence supporter described the general ways of being in Puerto Rico when discussing resistance against colonialism in her surroundings:

Those are things that one may think are stupid or small but they are not. Because when you live in a colony, what they want to do is erase your identity, (…). Speaking Spanish was illegal. Having a Puerto Rican flag was illegal. Singing our anthem was illegal. (…) So every act counts. And there's so many, that it's amazing to watch. I entertain myself looking for it, I swear. (*Participant 14*)

This participant highlights the intertwined relationship between symbolic acts with ways of being, identity, and resistance considering the history of repression and oppression. In line with definitions of everyday resistance as “small acts of resistance (…) done (…) informally through the culture of subordinated groups” (Johansson & Vinthagen, 2019, p. 26), the participant emphasizes that while these acts may look “small” and mundane, they are nevertheless meaningful and common forms of resistance (“there's so many”). She further emphasizes the influence of past repression of culture over peoples' identity and ways of being that make these acts important forms of resistance. Moreover, because they are embedded in everyday life, as the participant describes, they can be practised more widely, which may explain their perceived normativity.

### Staying on the land despite hardship and struggle

The quote discussed in the previous theme alludes to one of the struggles Puerto Ricans face, namely the physical displacement of Puerto Ricans through waves of migration to the US mainland. This has been primarily because of failing systems of governance and essential services, and the rising costs of living, which constitute the material, colonial realities of living in Puerto Rico (Capielo Rosario et al., 2023). Thus, as a 27‐year‐old female independence supporter who moved back to Puerto Rico after Hurricane María states: “living here is an act of resistance, staying here with how easy it is to leave” (*Participant 3*).

The ease of leaving she refers to is because of the US citizenship imposed upon Puerto Ricans. However, certain rights afforded to other US citizens do not apply to Puerto Rico residents (e.g., access to social security, voting rights). Thus, US citizenship brings some agency (e.g., freedom of movement) while also serving as a mechanism of colonial oppression (Font‐Guzmán, 2013). Social identity theory posits that the individual ability to leave the disadvantaged group to improve one's status should lead to individual mobility efforts and demobilize collective resistance (Haslam & Reicher, 2012; Tajfel & Turner, 1986). Consequently, US citizenship is a form of permeability that could decrease collective resistance among Puerto Ricans because they can leave the hardships of colonial oppression by moving to the US mainland. Thus, refusing to act upon this individual permeability is a theoretically interesting form of resistance.

Resistance through refusal to leave, mostly expressed by younger participants, is further motivated by the chat leak that triggered the mass protests of Summer 2019 to oust the Governor, where one chat member wrote: “It's so wonderful, there are no Puerto Ricans” (Valentín Ortiz & Minet, 2019). Participants often referred to this quote in explaining their motivation for this form of resistance. For example, a 29‐year‐old male independence supporter stated: “…we keep persisting, because they want to see here a Puerto Rico without Puerto Ricans” (*Participant 17*). This persistence in staying in Puerto Rico also implies resilience. For example, a 22‐year‐old woman independence supporter described sharing her experience of living in Puerto Rico with others outside of Puerto Rico:

In my presentation for [a conference], I remember that (…) they asked about our struggles, and I remember that when one lives here, you say “ah, it's fine”, but when others see it, their faces were like, “that's rough”, and I say it's true. I felt proud to say “yes, these things happen to us, and we are still here”. (*Participant 16*)

The hardships this participant refers to include a fragile infrastructure (e.g., power, utilities; see Bonilla, 2020) that was further debilitated by Hurricane María and the 2020 earthquakes, and Puerto Rico's heavily defunded, inaccessible public university system (Brusi & Godreau, 2019). While this participant addresses how these crises directly affect her, she also expresses pride in the resilience and endurance required to live in Puerto Rico. This conceptually aligns with the Palestinian notion of *sumud*, resilient resistance involving a determination to stay on the land despite enduring colonialism and occupation (Hammad & Tribe, 2021).

#### Staying on the land as a desired, future resistance strategy

Participants also described the importance of staying in Puerto Rico as a resistance strategy that should occur more. For example, a 23‐year‐old independence supporter describes her hope for collective resistance: “I know it's really tough, but trying to stay here, and see what can be done. Because if we stay waiting for someone else to do it, nothing's going to change” (*Participant 14*). This implies that Puerto Ricans need to stay to improve the conditions themselves, perhaps because of the local colonial governance's investment into Puerto Ricans' displacement. Therefore, the solution for Puerto Ricans to resist this displacement is to occupy space in the archipelago despite the struggles, and eventually reconfigure governance and the economy to prevent further displacement—as elaborated in the next theme.

### Prefigurative politics

Beyond the everyday resistance strategies represented in the first four themes, prefigurative politics, a form of collective action, was discussed as another way to resist colonial oppression. This strategy is characterized by individuals or groups directly taking actions outside of the existing structures to model on a smaller scale the society they want to see, thereby challenging dominant societal features (Boggs, 1977; Cornish et al., 2016; Dutt, 2018). These initiatives address systemic inequities perpetuated by the current structures. Moreover, these alternatives are not meant to plug the gaps in the system (which can help maintain unequal systems, a common critique of non‐profits; see Spade, 2020), but instead to empower citizens to fight for a system that meets all peoples' basic human needs (Ortiz Torres, 2020). Puerto Rican psychologists have discussed prefigurative politics efforts such as mutual aid in the aftermath of Hurricane María as *autogestión* and “sovereign acts” (Ortiz Torres, 2020; Serrano‐García, 2020). While our participants were not engaged in these efforts themselves, they described this resistance strategy as normative. Given the similarities across the two dimensions that were represented, here we only discuss desired, future resistance (see Appendix S2 for sample quotes of descriptive norms).

#### Prefigurative politics as a desired, future resistance strategy

The desired, future form of prefigurative politics that participants described focused on building sustainable agricultural resources outside of the official infrastructure. This would counter colonial oppression, considering 85% of the food is imported from the US mainland (Santiago‐Torres et al., 2019). For example, a 19‐year‐old woman independence supporter offered agricultural production as a path for future anti‐colonial resistance:

These economists come and try to give the narrative that well, we don't even have rice made in Puerto Rico. Yes it's true because since they buy so much from the US (…) then of course production here would stop. But you know, if we make everything here, (…) I think it's a necessary change for us to be us, and to not be controlled by any means by anyone, (…) but there needs to be more [agricultural] production in Puerto Rico. (*Participant 15*)

This participant counters the argument of food dependence elites use to justify colonialism, suggesting agricultural production as an anti‐colonial resistance strategy. She also suggests that this material anti‐colonial resistance strategy (building food production) could counter dependence by gaining control over resources, while serving identity‐based needs of preserving Puerto Ricans' ways of being (“for us to be us”)—which are both sites of colonial domination (Bulhan, 2015).

Prefigurative politics as future anti‐colonial resistance also involved participants' suggestion of developing and strengthening the local economy. For example, a 25‐year‐old male anti‐colonial supporter proposed utilizing lands designated for agricultural development by Puerto Ricans, in contrast to the local governance's investment in tourism. This would directly counter colonized nations' tendency to continue extractive economies even after independence (Quijano, 2000), and it offers an alternative practice for the future.

Interestingly, participants often reflected on prefigurative politics through more individual means, despite it being a form of collective action (Cornish et al., 2016). For example, a 19‐year‐old woman independence supporter suggested “more [Puerto Ricans] should do agricultural work”, without discussing how it could occur collectively (*Participant 15*). Similarly, a 27‐year‐old woman independence supporter said that “if action is not taking place in a collective manner, (…) make it your life project or even in things that seem silly but aren't like supporting local businesses, (…) buying local products, (…) buying land” (*Participant 3*). This evokes definitions of everyday resistance, which is mundane and occurs without formal organization (Johansson & Vinthagen, 2019; Rosales & Langhout, 2020), while also implying that anti‐colonial resistance involves developing a local economy and taking ownership of the land. Furthermore, this participant suggests one reason for why individual strategies prevail over collective strategies—because action is currently not occurring collectively, people may take up resistance through individual means until such a movement emerges (see Orazani & Teymoori, 2024).

### Protests

Adding to this point about the current lack of anti‐colonial collective action, only few participants mentioned conventional protests as a resistance strategy. Moreover, this theme was only represented as a future desired resistance strategy, and not as a present, personal strategy or resistance norm. Notably, protests do occur for other societal grievances. For example, in describing which resistance strategies against colonialism could be tried in the future, a 23‐year‐old woman independence supporter describes the success of other protests:

I'd say yes, we saw it, for example, when Summer 2019 happened, we saw. It was historic, from how much it moved people, (…). But definitely, we have the power to do something. If we set our minds to it, (…) of course. (*Participant 14*)

This quote suggests that this participant perceives a sense of collective efficacy (van Zomeren et al., 2008), referring to the mass protests to oust governor Rosselló to reason why more prototypical collective action also could be used in the future to resist colonialism. However, this participant also suggests that the collective effort needed for this is currently not yet actualized (“if we set our minds to it”). A 19‐year‐old woman independence supporter mentioned protests as one of several strategies that should occur alongside other resistance strategies: “Protesting, having forums where people can learn and all about what's happening, (…), educate, I think that's most important” (*Participant 15*). Notably, despite some scarce discussion of protests, more participants rejected this strategy as a means to resist colonial oppression. While the reasons for rejecting certain resistance strategies are beyond the scope of this paper, future research should examine why people reject protests in certain contexts, such as due to risk of repression.

## DISCUSSION

### Summary and theoretical contributions

The present study provides a qualitative investigation of the anti‐colonial resistance strategies Puerto Ricans discussed regarding the current colonial arrangement with the United States. We identified six distinct anti‐colonial resistance strategies that mostly include forms of everyday resistance (psychological resistance, cultural and symbolic resistance, critical consciousness‐raising, and staying on the land despite hardship). Additionally, participants discussed two forms of collective action (prefigurative politics and protests), but these were less common, and not resistance strategies they personally engage in.

While social psychologists have extensively addressed collective action, these findings therefore indicate that there are many resistance strategies beyond prototypical collective action that are important to consider. These findings offer several empirical and theoretical contributions to the social psychology of resistance. Resistance strategies beyond collective action have been studied in other fields such as sociology and anthropology (e.g., Scott, 1985; Johansson & Vinthagen, 2019), and there is some theoretical discussion of them in social psychology (e.g., Leach & Livingstone, 2015; Rosales & Langhout, 2020; Vollhardt et al., 2020), but very little empirical work investigating them. Consequently, the present findings identify understudied forms of resistance, show that several different forms of resistance occur simultaneously, and that collective action is not widespread or preferred in this context. This is important for several reasons: First, it helps prevent misconstruing lack of protest and other commonly studied forms of overt, organized collective action in colonial oppression contexts as passivity, lack of agency among oppressed groups, or as internalized oppression (see also Rosales & Langhout, 2020). Second, it helps integrate knowledge from other disciplines to broaden our conceptual understanding of resistance, and in turn contributes insights and theoretical explanations from social psychology (e.g., regarding identity processes) to this literature. Third, it lays the foundation for future theorizing about the psychology of resistance, and how different resistance strategies relate to eachother. By theorizing beyond collective action strategies, social psychology can enrich our understanding of the psychology of resistance by studying multiple resistance strategies, how they may influence eachother, are shaped by the context, and contribute to social change with different social change goals (Sweetman et al., 2019). We discuss more specific contributions of these findings and resulting future research directions in the following.

First, the range of resistance strategies we identified can be classified along different theoretical dimensions. One way in which these strategies vary is in their intended location of social change. For example, psychological resistance as well as symbolic and cultural resistance focus change efforts on the disadvantaged group's identity (identity‐focused strategies, see Figure 1), while critical consciousness‐raising, staying despite hardship and struggle, prefigurative politics, and protests tackle specific problems that colonialism creates (e.g., lack of critical awareness, displacement, failure of official systems). Notably, these dimensions are not mutually exclusive because colonial oppression also targets identity and all aspects of daily life and being (Bulhan, 2015; David, 2014; Maldonado‐Torres, 2007). Thus, the current findings suggest that in such contexts where so many aspects of one's collective and individual life are affected by long‐term oppression, such as under colonialism—as opposed to more commonly studied contexts in the collective action literature that focus on more circumscribed grievances with a more sudden onset—forms of resistance about identity, other symbolic aspects, and everyday being may be particularly relevant.

Second, another theoretical dimension along which these resistance strategies vary is whether they operate on the individual or collective level. Whereas psychological resistance focuses on individuals determining the meaning of group disadvantage for themselves (Leach & Livingstone, 2015), the other five resistance strategies we identified also or solely operate at the collective level. For example, critical consciousness‐raising aims to address the collective through fostering critical awareness in other group members. Furthermore, while participants discussed their potential engagement in prefigurative politics, the literature describes this as a form of collective action to create smaller scale alternative structures in society (Cornish et al., 2016). These findings suggest that the line between individual and collective resistance is often blurred in this context, which again makes sense given the pervasive nature of colonialism that people are resisting at multiple levels (Bulhan, 2015). These findings therefore also challenge and expand theorizing about collective action and resistance in the social psychological literature. For example, two of the anti‐colonial resistance strategies we identified raise important questions from the perspective of social identity theories of collective action (e.g., Haslam & Reicher, 2012; Tajfel & Turner, 1986; van Zomeren et al., 2008). Psychological resistance (Leach & Livingstone, 2015) was discussed by participants but can also be viewed as social creativity, which is often juxtaposed with challenging the status quo through collective resistance (Tajfel & Turner, 1986). This resistance strategy was also the only theme that included quotes from two status quo or statehood supporters (i.e., not inclined towards resistance against the system; see Rivera Pichardo et al., 2022). This theoretical dilemma warrants attention when considering the differences between individuals' lay theories of resistance and academic conceptualizations of resistance—which vary across contexts considering the oppression faced. In this case, we argue that psychological resistance is an important site of resistance in colonial contexts, given the effects of coloniality on identity (Bulhan, 2015). Moreover, it could serve to prepare for future collective action mobilization (Orazani & Teymoori, 2023). However, if this psychological process instead serves to minimize or deny oppression, it could also demobilize potential collective action engagement (Becker, 2012). Therefore, while our interview questions did not allow for examining the relationships between different resistance strategies, future research should address how individual and collective resistance strategies relate to each other. Similarly, the context‐specific resistance strategy of staying on the land also challenges predictions of permeability in social identity theory (Tajfel & Turner, 1986). Since US citizenship affords Puerto Ricans permeability to move to the US mainland and leave the hardships of colonial oppression, the choice to reject this individual mobility is significant and turns this seemingly individual choice into a response to a collective grievance. Therefore, this finding also implies the importance of a context‐centered approach to studying resistance.

Third, our analysis sheds light on the importance of distinguishing between different levels of resistance. While social psychological theories suggest that perceived group norms and individuals' resistance actions should be linked (e.g., Penić et al., 2024; van Zomeren et al., 2004) and willingness for future action is often used as a measure of engagement in collective action (e.g., Stewart, 2017; see Uluğ et al., 2022 for a critique), our findings highlight many discrepancies between the resistance strategies participants personally engage in, perceived norms of resistance strategies that other ingroup members engage in, and which strategies they desire for the future (see legend in Figure 1). Specifically, four resistance strategies showed such discrepancies: psychological resistance (only discussed as a personal resistance strategy, presumably due to its individual and less observable nature), staying despite hardship and struggle (discussed as a personal and desired future resistance strategy, but not as a norm), prefigurative politics (described as both a normative and a desired future strategy, but not as a personal resistance strategy), and protests (described only as a desired future strategy, and not as a normative or a personal resistance strategy). These discrepancies raise questions for future research. For example, for which resistance strategies do perceived resistance norms influence peoples' individual choices of resistance, and for which may norms be less relevant? Moreover, to what extent do efficacious actions the ingroup has taken in the past shape which resistance strategies are desired for future steps towards liberation (e.g., Freel & Bilali, 2022), or alternatively informed by the ingroup's tactical mistakes or what has been tried but not been successful due to repression (e.g., Ünal & Coşkan, 2023)? Notably, because our interview questions led to unprompted responses, where we did not ask participants to address the degree to which each resistance strategy they mentioned operated on each level (personal, group norms, future strategy), these findings need to be interpreted with caution, and future research should address these questions systematically.

### Strengths and limitations

The current study has several strengths. First, it is an empirical investigation of understudied resistance strategies in a colonial context that is underrepresented in social psychology, like the so‐called non‐WEIRD contexts in the Global South more generally (Bou Zeineddine et al., 2022; Rad et al., 2018). Another strength is the qualitative methodology, allowing participants to define for themselves what they considered to be anti‐colonial resistance without prompting any specific strategies. This allowed us to identify resistance strategies that are often excluded in quantitative measures of resistance. For example, the frequency of critical consciousness‐raising participants discussed may indicate more concerted resistance efforts that may have been missed in more commonly used measures of collective action, which may underestimate agency and resistance in Puerto Rico. By including interview questions about perceived norms and future resistance strategies in addition to participants' own actions, we were able to gain a more complete understanding of the psychology of resistance, shaped by current group norms and hopes for the future. Furthermore, another strength is that it addresses a broader spectrum of possible dimensions of resistance and how they may work together in a given context (see Vollhardt et al., 2020).

The current study also had some limitations. Despite efforts to recruit a more diverse sample in terms of social class, our participants were highly educated, which may explain the prevalence of critical consciousness‐raising. With a more diverse sample (e.g., older, less educated), different resistance strategies may have been identified. Likewise, had we explicitly recruited political activists, rather than a more general community sample as we did on purpose to examine more commonly pursued resistance strategies, our findings may have also included more examples of collective action. Similarly, several of these strategies may also be unique to colonial contexts (e.g., staying on the land) in comparison with other strategies given the nature of colonial oppression (Bulhan, 2015). As resistance involves countering destructive power, it would make sense for strategies to adapt upon the nature of the destructive power in itself, and the present state of oppression. In this case, prototypical collective action may not be a salient strategy to engage in in the present to resist colonialism, while other strategies were more relevant. Therefore, future research would do well to consider the nature of the destructive power at play in order to discern the tactics that groups would consider most appropriate or useful to engage in resistance.

While it was a strength that participants discussed resistance strategies they found meaningful, without being prompted to address specific ones, we cannot systematically compare participants' responses or rule out that they may have also agreed with strategies they simply did not think to bring up. For example, the lack of discussion of personal engagement in prefigurative politics by participants is counter to the vast documentation of this strategy by Puerto Rican community psychologists (Miranda Gierbolini et al., 2022; Ortiz Torres, 2020; Serrano‐García, 2020), such as mutual aid networks (e.g., Red de Apoyo Mutuo), food pantries (e.g., Comedores Sociales de Puerto Rico), community gardens (e.g., Huerto Comunitario San Mateo), or solar generation networks (e.g., Casa Pueblo). Likewise, there are organizations that do engage in protests to address issues caused by colonial oppression (e.g., Campamento Carey; Colectiva Feminista en Construcción). Future research would benefit from more targeted questions based on our findings (e.g., whether prefigurative politics is understood as an individual strategy demobilizes engagement in more collective efforts).

## CONCLUSION

In summary, the present findings expand on the complex nature of varied anti‐colonial resistance strategies shared by participants in a present‐day colonial context. Beyond more commonly studied forms of resistance, participants discussed a range of everyday resistance strategies and collective action while they only reported engaging in everyday resistance strategies. The resistance strategies participants discussed varied by the intended location of change, the actor, and with regard to whether they were described as participants' own actions, as group norms, or as a desired future resistance strategy. Notably, most of these resistance strategies occur at the individual and interpersonal levels, rather than more large‐scale, organized collective efforts. Therefore, a perceived lack of overt mass mobilization to resist oppression may not always mean internalized oppression and system justification. Instead, these findings demonstrate the need to consider the historical and political conditions that could (de)mobilize certain resistance strategies, and the resources groups have—including group norms—that enable other resistance strategies. Furthermore, the findings highlight the importance for social psychology to consider multiple kinds of resistance strategies, and we invite future research to consider a full range of resistance strategies, as well as explore the relationships these strategies have with eachother. Overall, these findings shed light on anti‐colonial resistance in Puerto Rico, where even despite a history of repression against independence supporters, as well as the immense political power of the statehood movement in local governance, hope for liberation lives on.

## AUTHOR CONTRIBUTIONS

**Carmen Marazzi:** Conceptualization; methodology; formal analysis; writing – original draft; writing – review and editing; investigation; funding acquisition. **Johanna Ray Vollhardt:** Methodology; conceptualization; formal analysis; writing – review and editing; investigation; funding acquisition.

## CONFLICT OF INTEREST STATEMENT

All the authors have no known conflicts of interest to disclose.

## Supporting information


Appendices S1–S2.

## Data Availability

Because of the qualitative and potentially sensitive nature of the data, we did not make the data publicly available in a data repository. However, the data extracts analysed in this paper are available from the corresponding author upon reasonable request.

## Footnotes

^1^
The Fiscal Control Board is the product of 2016 US federal law PROMESA, where US government appointed officials are given the right to control Puerto Rico's local government and finances, including veto power over local legislation. This shattered any semblance of autonomy Puerto Rico had reconstructured a direct colonial relationship (Fortuño Bernier, 2017).
