# Supplementary material for: “When you live in a colony… every act counts”: Exploring engagement in and perceptions of diverse anti‐colonial resistance strategies in Puerto Rico
Source: Br J Soc Psychol. 2024 Oct 9;64(2):e12808. doi: 10.1111/bjso.12808 (PMC11927381; doi:10.1111/bjso.12808)
Supplement: Supplementary file 1 — Appendices S1–S2. [file BJSO-64-0-s001.docx]

**Appendix S1: Full Interview Schedule Provided**

| **In English (translation)** | **In Spanish (original version used in interview)** |
| --- | --- |
| - Would you like to introduce yourself, tell me a little bit about yourself? - POTENTIAL FOLLOW UPS:   - How old are you now?   - What do you do for a living?   - Which city/town are you from?   - Living in the US     - Have you spent time living in the US? How long?     - How long have you lived in Puerto Rico?     - Do you have family in the US?   - Activism     - Do you consider yourself an activist?     - Do you have family members who are/were activists?  1. Can you describe, briefly, what it is like being Puerto Rican?    1. How important is it to you that you are Puerto Rican? 2. Can you describe the relationship Puerto Rico has with the United States?    1. What would you call it?    2. What would others call it? What do you think about those terms? [do you agree or disagree, and why?] 3. Do you believe that (2a response) is a problem for Puerto Rico/Puerto Ricans?    1. Why? Why not?    2. How do you think this issue will develop/evolve/change over time?    3. IF NO, do you believe there is another problem that’s more important?       1. Which one? Why?       2. Does (2a response) have anything to do with this problem, or is it a separate issue? Why/why not? How? 4. Is (2a response) important/relevant/ pertinent to you personally?    1. To your identity as a Puerto Rican?    2. To your daily life?    3. To your family and your family’s history? 5. Why do you believe (2a response) happened?    1. Are there any events or policies in the past that you recall that are particularly important for understanding why and how (2a response) happened? 6. Who do you believe is responsible for (2a response)?    1. How responsible is the US government for (2a response)?    2. Do you think that we, as Puerto Ricans, are responsible or not for (2a response)? 7. Do you think the situation Puerto Rico is in is right or justified, or not?    1. Why, why not? 8. How do you feel when you think about (2a response)?    1. Do you have hope?    2. Do you feel prideful?    3. Do you feel ashamed?    4. Do you have any anger? At what/whom? 9. What should ideally be done to address the problem of (2a response)?    1. What do we need in order to do that?    2. Do we have what we need to do that, or are there things we don’t have in order to get there?    3. Do you believe we can and could address (2a response)    4. Do you believe we have the power to change (2a response)? 10. What are the ways in which people in Puerto Rico show opposition to (2a response)?     1. Do you think these strategies have been effective?     2. (If not already implied): who is using these strategies?     3. What else do you think we should try?     4. All things considered (repeat what they said), what do you think should be our main goal to focus on right now? 11. And what do you think should be our long-term, ultimate goal?   **Demographics**   - Gender- How would you describe your gender identification? - Race- In your own words, how would you describe your race/racial identity? - How would you describe your social class? - What is the highest level of education that you have achieved? - How would you describe your political orientation? (follow up: left-right)   Thank you so much for taking the time to do this interview with us! Our last question is: Are there any questions we should have asked in the interview but didn’t ask about? Is there anything else related to these topics that you want to share with us that we haven’t yet talked about? | - ¿Me podrías introducirse y decir un poco sobre usted? - POTENTIAL FOLLOW UPS:   - ¿Cuántos años tienes?   - ¿A qué te dedicas?   - ¿De qué pueblo eres?   - Viviendo en EEUU     - ¿Has vivido por algún tiempo en los Estados Unidos? ¿Por cuánto tiempo?     - ¿Por cuánto tiempo has vivido en Puerto Rico?     - ¿Usted tiene familia en los EEUU?   - Activismo     - ¿Usted se considera un activista?     - ¿Tienes familiares quiénes son o fueron activistas?  1. ¿Me podrías describir brevemente lo que es para ti ser puertorriqueño?    1. ¿Cuán importante es para usted ser puertorriqueño? 2. ¿Me podrías describir la relación que tiene Puerto Rico con los EEUU?    1. ¿Cómo lo llamarías?    2. ¿Cómo otras personas llamarían esta relación? ¿Qué piensas cuando oyes estos otros términos? [¿estás de acuerdo o en desacuerdo, por qué?] 3. ¿Usted cree que (2a response) es un problema para Puerto Rico/para los puertorriqueños?    1. ¿Por qué? ¿Por qué no?    2. ¿Cómo crees que este asunto podría desarrollar/evolucionar/cambiar con el tiempo?    3. SI NO, ¿crees que hay otro problema que es más importante?       1. ¿Cuáles? ¿Por qué?       2. ¿Crees que el/la (2a response) tiene algo que ver con este problema, o es un asunto separado? ¿Por qué? ¿Por qué no? ¿Cómo? 4. ¿Es (2a response) importante/relevante/ pertinente a usted personalmente?    1. ¿A su identidad como puertorriqueño?    2. ¿A su vida diaria?    3. ¿Para su familia o a la historia de su familia? 5. ¿Por qué crees que (2a response) ocurrió?    1. ¿Hay algún evento o ley/política pública en el pasado que sea particularmente importante para entender por qué y cómo (2a response) ocurrió? 6. ¿Quién crees que es responsable para (2a response)?    1. ¿Cuán responsable es el gobierno estadounidense por (2a response)?    2. ¿Crees que nosotros, como puertorriqueños, somos responsables o no por (2a response)? 7. ¿Crees que la situación en que se encuentra Puerto Rico es correcta o justificada, o no?    1. ¿Por qué? ¿Por qué no? 8. ¿Qué sientes o qué emociones tienes cuando piensas en (2a response)?    1. ¿Sientes esperanza/ilusión/fé?    2. ¿Sientes orgullo?    3. ¿Te da vergüenza o bochorno?    4. ¿Te enoja? ¿A qué/quién? 9. ¿Qué es lo que se idealmente debe hacer para abordar con el problema de (2a response)?    1. ¿Qué necesitamos para poder realizar esto?    2. ¿Tenemos los recursos para poder hacer esto, o hay recursos que no tenemos para abordarlo/resolverlo?    3. ¿Crees que tenemos la voluntad y capacidad de abordar/resolver (2a response)    4. ¿Crees que tenemos el poder de abordar o resolver (2a response)? 10. ¿Cuáles son las formas/maneras/ estrategias en que los puertorriqueños enseñan su oposición a (2a response)?     1. ¿Crees que estas estrategias han sido efectivas?     2. (If not already implied): ¿quiénes están usando estas estrategias?     3. ¿Qué otras formas/estrategias crees que deberemos probar?     4. Considerando todo (repeat what they said), ¿cuál debería ser nuestra meta para enfocarnos en este momento? 11. ¿Cuál debería ser nuestra meta a largo plazo?   **Demographics**   - ¿Cómo describirías su género/su identidad de género? - En tus propias palabras, ¿cómo describirías su identidad racial/su raza? - ¿Cómo describirías su clase social/ económica? - ¿Cuál es el nivel más alto de educación que has logrado? - ¿Cómo describirías su orientación política? (follow up: izquierda-derecha)   ¡Muchísimas gracias por tomar el tiempo de su vida para realizar esta entrevista con nosotros! Tengo una última pregunta: ¿Hay algunas preguntas en que debíamos haberle preguntado en esta entrevista que no hemos hecho? ¿Hay alguna otra cosa con relación a estos temas que quieres compartir con nosotros que no hemos tocado/hablado? |

**Appendix S2: Additional Proof Quotes for All Themes and Dimensions, with Spanish original quotes and English translation by the first author**

| **Psychological resistance** | | |
| --- | --- | --- |
| 1. Personal resistance strategies | “I don’t want to have that feeling of, […] resentment towards what is being redefined as being Puerto Rican because there's always those who say "Oh no, Puerto Ricans are lazy or don't want to work, or they behave very violently, are very loud," and it's like, we're not just that. I mean, ask who is defining us and why” (participant 14) | “No quiero tener ese sentimiento de […] resentimiento hacia lo que se está redefiniendo como es ser puertorriqueño porque siempre están lo que dicen “ah no, los puertorriqueños son vagos o no quieren trabajar, o se comportan bien violentos, son bien loud y es como que, we’re not just that. Osea, preguntar quién nos está definiendo y por qué.” (participant 14) |
| **Critical consciousness-raising** | | |
| 1. Personal resistance strategies | “The only thing I believe is, personally, what I can do is educate, educate, educate, question, question people. Sometimes it frustrates me because there are people that you question, and they have no argument, they have nothing to say, but also don’t think nor open themselves up to think. But at least create a reasonable doubt, and always when I have the opportunity to create that reasonable doubt I do it" (participant 4). | “Yo lo único que creo es, personalmente, lo que yo puedo hacer es educar, educar, educar, cuestionar, cuestionar a la gente. A veces me frustro porque hay gente que tu cuestionas, y no tienen argumento, no tienen nada que decir, pero tampoco piensan ni se abren para pensar, pero por lo menos crear una duda razonable, y siempre que yo tengo la oportunidad de crear esa duda razonable aprovecho” (participant 4) |
| 1. Descriptive resistance norms | “There are several people who are being very vocal and really active and that create a collective consciousness and also to younger people, who understand that it is not a matter of taking out our flag, and ‘yes, my country is beautiful, Puerto Rico’, but that it has to be activism, and action towards that.” (participant 2) | “Hay varias personas que están siendo bien vocales y bien activistas y eso crea una conciencia colectiva y también a la gente más joven, que entienda que no es cuestión de sacar la banderita y, ‘si sí mi país es lindo, Puerto Rico’, sino que tiene que haber un activismo, y acción hacia eso.” (participant 2) |
| 1. Future, desired resistance | “I simply know that for the moment I have to continue studying, that I have this degree that I understand has some type of useful application eventually, I will continue working for […] in their projects even though I am not a journalist, I feel that nothing more that the mere fact of just working there and being able to support people who are doing such important things because that makes me feel that I have value or what I do has value. I think one can propose it for themselves, maybe the best way is to work at the individual level.” (participant 3) | “yo simplemente sé que yo tengo que por el momento tengo que seguir estudiando qué tengo esta carrera que yo entiendo que tiene algún tipo de aplicación útil eventualmente, sigo trabajando para el […] en sus proyectos y aunque no soy periodista siento que nada más con el mero hecho de trabajar ahí y poder brindarle apoyo a personas que están haciendo cosas tan importantes pues eso a mí me hace sentir que lo que yo hago tengo valor o tiene valor y yo creo que uno se lo puede plantear, quizás la mejor manera es planteárselo de una forma individual. (participant 3) |
| **Symbolic and cultural resistance** | | |
| 1. Personal resistance strategies | “The cultural relationship between the United States and Puerto Rico is also one of oppression to because it claims at the end of the day, what's listened to today? Pop music. That, Bad Bunny, yes we listen to Bad Bunny because Bad Bunny became popular in the US with the US music industry, […] Salsa is not listened to anymore. There are many people who culturally are like, ‘Ugh, you listen to this? If you are young?’ and yes, I listen to it, because it's my roots, but that's what it is, culturally in general, everything is summed up with how the United States oppresses Puerto Rico.” (participant 20) | “La relación cultural de Estados Unidos con Puerto Rico es de opresión también porque lo que reivindica a fin de cuentas, ¿qué se escucha actualmente? Música pop. Esto Bad Bunny, sí se escucha Bad Bunny es porque Bad Bunny se hizo popular en Estados Unidos con la industria musical estadounidense. […] La salsa ya no se escucha. Hay mucha gente que culturalmente como que, "Uy, ¿tú escuchas eso? ¿si tú eres joven?" Y sí. Yo lo escucho, son mis raíces, pero eso es lo que hay, culturalmente en general, todo se resume con que Estados Unidos oprima Puerto Rico.” (participant 20) |
| 1. Descriptive resistance norms | “I believe that there is a very strong resistance from the cultural world, because it's in the documentation in the records of our different cultural manifestations, or it’s the persistence to maintain our dynamic identity as Caribbean, Puerto Rico, is what makes us speaking Spanish a form of resistance.” (participant 2) | “Yo creo que hay una resistencia bien fuerte del mundo cultural. Porque está la cuestión de documentación en registro en las distintas manifestaciones cultural, o viene esa persistencia de mantener esa identidad dinámica del Caribe, Puerto Rico, el que hace que hablemos español son una forma de resistencia.”  (participant 2) |
| 1. Future, desired resistance | “Right now I think it is a moral imperative for anyone to assume their Puerto Ricanness and understand what that really means, and look at that from here on the short term.” (participant 3) | “Ahora mismo creo que es imperativo moral de cualquier persona asumir su puertorriqueñidad y entender lo que eso de verdad significa y mirarlo de aquí a corto plazo.” (participant 3) |
| **Staying despite hardship and struggle** | | |
| 1. Personal resistance strategies | “I would like to improve the situation on the island because there are already many bad things on the island. This and also there is a feeling of frustration with everything that's happening on the island because it's difficult. What can I do being 21 years old? […] and what they are doing is displacing the Puerto Rican people. […] In short, it is a little conflictive. But I do have this desire to stay, I want to stay. (participant 20) | “Quisiera mejorar la situación en la isla porque ya hay muchas cosas malas en la isla. Esto pero también hay un sentido de frustración con todo lo que está sucediendo en la isla porque es difícil. ¿Esto que yo puedo hacer con 21 años? […] y lo que están haciendo es desplazar a la población puertorriqueña. […] En resumen, como que. Un poco conflictiva. Pero sí tengo como que el anhelo de quedarme, me gustaría quedarme. (participant 20) |
| 1. Future, desired resistance | “in the long term we can be owners of our own land, that the land belongs to the country,” (participant 3) | “a largo plazo el que podamos ser dueños de nuestra propia tierra, que la tierra le pertenezca al país” (participant 3) |
| **Prefigurative politics** | | |
| 1. Descriptive resistance norms | “they sold the land, and now my grandfather kept his house and a farm. But he can’t work on the farm because he’s messed up, so he sows on the roof. […] He planted about twenty things on the roof, and he sold them all. And today? Everything is sold. […] My grandmother, who has never worked outside the house, makes homemade sofrito with her ingredients. They don’t last two days in the refrigerator. Well, people come out of nowhere, ‘I want this much sofrito’. So in short, start small. We have many ways of being self-sustaining individually” (participant 14) | “fueron vendiendo los terrenos, y ahora mi abuelo se quedó con su casa, y una finca. Pero él no puede trabajar en la finca porque está jodío. Siembra en el techo. […] el sembraba sobre veinte cosas en el techo, y todas las vende. ¿Al sol de hoy? Todo se vende. […] Abuela, que nunca ha trabajado fuera de la casa, con los ingredientes hace sofrito homemade. No duran dos días en la nevera. Pues la gente de la nada llega, “quiero tanto de sofrito”. So, en definitiva, empezar pequeño. Nosotros tenemos muchas maneras para ser autosustentable, de manera individual.” (participant 14) |
| 1. Future, desired resistance | “One problem that Las Marías has is that there cannot have a lot of industrial development because most of the land in Las Marías is protected for agricultural uses because of its ecological value […] But they are not being developed, the majority are not being used. Therefore, it is not being use for the benefit of the people here. What’s more, not even to export abroad. And yet we see millions of dollars invested in the tourism industry, in the economy of the visitor, […] that is, luxuries when the majority of the people in Puerto Rico live below the poverty line and it's something that is more accessible to people outside than even to some people here. […] In other words, we have the tools, but we are not using them, and we are using ineffective tools because we think that this is the model that must be followed, because it is perhaps pleasing other political powers or political interests.” (participant 19) | “Un problema que tienen Las Marías es que no se puede hacer mucho desarrollo industrial porque la mayoría de los terrenos en Las Marías están protegidos por usos de agricultura, por su valor ecológico [...] Pero no se están desarrollando, no se están usando a la mayoría. Por consiguiente, no se le está utilizando para su beneficio para las personas de aquí. Es más, ni siquiera para exportar para fuera. Y sin embargo vemos millones de dólares invertidos en la industria del turismo, en la economía del visitante, [...], O sea, lujos cuando la mayoría de las personas en Puerto Rico viven por abajo del nivel de la pobreza y es algo que es más accesible para las personas de afuera que hasta para algunas personas de aquí. [...] O sea, tenemos las herramientas, pero no la estamos usando, y estamos usando unas herramientas inefectivas porque pensamos que ese es el modelo que hay que seguir, porque está complaciendo quizás a otros poderes políticos o intereses políticos,” (participant 19) |
| **Protests** | | |
| Future, desired resistance | "I think the most notable case is the one of Vieques and Culebra, where the Puerto Ricans demanded the departure of the US Navy in Vieques and that is a clear example of the power of the people against the treatment of the Americans because in the end they left these spaces, it took time, and they took people prisoner. They left Vieques and Culebra, (…) I believe that if the entire country agreed we could also resolve it just like what happened in Vieques and Culebra” (participant 17) | “Creo que el caso más notable es el de Vieques y Culebra, donde los puertorriqueños solicitaron la salida de la Marina en Vieques y eso es un claro ejemplo del poder del pueblo contra el trato estadounidense porque al final salieron de estos espacios, tardaron tiempo, cogieron gente presa. Salieron de Vieques y Culebra, (…) yo creo que el país completo si se pusiera de acuerdo pudiéramos resolverlo también igual que pasó en Vieques y Culebra” (participant 17) |
